# Brushing With Charcoal Toothpastes on the Surface Properties of a Giomer: A Comparative In-Vitro Study

**DOI:** 10.7759/cureus.86847

**Published:** 2025-06-27

**Authors:** Shivaughn Marchan, Kelee Bascombe, Amrita Rajhbeharrysingh

**Affiliations:** 1 Dentistry, The University of the West Indies, St. Augustine, TTO; 2 Restorative Dentistry, The University of the West Indies, St. Augustine, TTO

**Keywords:** charcoal-containing toothpastes, giomer, microhardness, nano-composites, surface roughness

## Abstract

Objective

This study aimed to quantify changes in the microhardness (VHN) and roughness (Ra) in three esthetic restorative materials: a giomer, a universal nano-hybrid, and a nano-filled composite after one and six months of simulated brushing with various types of charcoal-containing toothpastes.

Materials and methods

Forty specimens (8 mm x 8 mm x 2mm) of each material were fabricated using the manufacturer's instructions and randomized into five groups. Baseline VHN and Ra readings were taken. Simulated brushing took place with four charcoal-containing toothpastes. A non-charcoal-containing toothpaste was used as a positive control. VHN and Ra were then measured after one month and six months of simulated brushing. Statistical analysis was performed using a two-way repeated-measure ANOVA to assess whether the factors of time, toothpaste, or the interaction produced significant changes in VHN and Ra. Post-hoc Bonferroni tests were used for pairwise comparisons of material properties brushed with the various toothpastes.

Results

All tested materials demonstrated a general trend of increasing microhardness and decreasing roughness, or roughness that was not statistically significant from baseline. Interaction of time*toothpaste produced statistically significantly higher microhardness values for the giomer (p=0.003) and nanohybrid (p=0.023) materials. The roughness of the nanofilled material was significantly lower than baseline, with the interaction of time*toothpaste (p=0.014).

Conclusion

The factors of time, toothpaste, or the interaction of time and toothpaste produced significantly different Ra and VHN values; however, variations in the properties were deemed favorable.

## Introduction

Giomers are a relatively new class of restorative material with bio-active properties. Giomers have been described as a hybrid material between dental composite and glass ionomers, maximizing the esthetic and bioactive properties of these materials, respectively [1]. Giomers are a special class of composites that provide both protection against caries and functional and esthetic properties by incorporating particles of pre-reacted glass filler into a resin matrix of traditional principal monomers used in dental composite materials [1]. Giomer has been compared for color and surface roughness to other bioactive restoratives such as glass ionomer and resin-modified glass ionomer and has shown superior values for both these tested parameters compared to other bioactive restoratives [2].

Microhardness, the resistance of a material to resist indentation and the combined product of the measure of polymerization and filler loading by volume, is an important surface property of several dental restoratives [3,4]. Microhardness measurements indicate a material’s property to resist abrasive wear from mastication or toothbrushing. Beautiful Plus is a giomer that, when evaluated for microhardness, demonstrated values well above those of other bioactive materials [5]. Beautiful II, a packable giomer, has been shown to have similar microhardness values compared to nano-hybrid composite resin before immersion in water or various beverages [6].

Surface roughness of restorations can influence both biofilm formation and color stability of esthetic restorative materials [7,8]. Kilic and Gok have demonstrated that the surface roughness of giomers can be comparable to composite resin; however, this is dependent on the type of finishing system used [9]. Toothbrushing can influence surface roughness [10,11] and microhardness of esthetic restoratives [12]. Whitening toothpastes have been shown to cause significant increases in both surface roughness and microhardness of esthetic restorative materials compared to those brushed with non-whitening toothpastes [12].

Over-the-counter charcoal-containing toothpastes have recently gained popularity as whitening toothpastes [13]. This study aimed to quantify changes in the microhardness and roughness of a giomer compared to a nano-hybrid and a nano-filled composite after one and six months of simulated brushing with various types of charcoal-containing toothpastes. The null hypothesis stated that there would be no difference in the mean values of microhardness and roughness for any of the tested materials because of time, toothpaste, or the interaction of time and toothpaste.

## Materials and methods

Three esthetic restoratives, a giomer (Beautiful II, Shofu), a nano-filled (Z350, 3M), and a nano-hybrid (Z250, 3M) in shade A3 were used in this study. The toothpastes used in this study, together with the manufacturers' details, are shown in Table 1.

**Table 1:** Toothpastes used together with the manufacturer's details

Toothpaste	Manufacturer
Optic White	Colgate-Palmolive Company New York, USA
Pursito	Jarosa Inc, Boyne City, Michigan, USA
Crest 3D with Charcoal	Procter & Gamble Company, Ohio, USA
Cuprapox	Curaden USA Inc, Arizona, USA
Colgate Essentials with Charcoal	Colgate-Palmolive Company New York, USA

A non-charcoal containing whitening toothpaste, Optic White (Colgate Palmolive), was used as a positive control.

A power analysis (G Power 3.0.10, Universitat Kiel, Germany) determined 40 specimens of each esthetic restorative were needed given an effect size of 0.5, a power of 0.8, and a total of five groups for each tested restorative. Mylar-covered specimens were fabricated for each material using split Teflon-coated stainless-steel molds (8 mm x 8mm x 2mm) and polymerized with an LED curing light in a standard mode (Valo, Ultradent, South Jordan, Utah) in an overlapping motion for 20 seconds. The intensity of the LED light was measured at 978 mW/cm^2^ using a radiometer (Demetron, Kerr Corporation, Brea, California). Following polymerization according to the manufacturers’ instructions, the specimens were polished and stored at 100% humidity in a light-proof container until baseline measurements of microhardness and roughness were taken. Unidirectional polishing was accomplished with Super-Snap discs (Shofu, San Marcos, California) in decreasing coarseness from coarse to super fine for ten seconds each. One set of discs was used for every three specimens. Specimens were mounted in cylinders of auto-polymerizing clear acrylic resin to facilitate toothbrushing. Specimens from each esthetic restorative were numbered at the bottom of the acrylic resin and randomly assigned to one of the five toothpaste groups using a randomizing application (randomizer.org). Baseline measurements for roughness (Ra) and Vickers microhardness (VHV) were taken. 

Surface roughness testing

Immediately following polishing, the giomer or composite specimens were quantitatively assessed by profilometry using the Mitutoyo Surftest 401 surface roughness analyzer (Mitutoyo America Corporation, Aurora, Illinois) with a cutoff of 0.25 mm, a transverse length of 1.6 mm, a sample length of 1.25 mm, and a vertical bandwidth of 50 µm. The stylus used a 15mN force at a speed of 5.08mm/sec for each tracing. Three parallel tracings were used to scan the finished surface of each specimen with a side shift of 1.5 mm between tracings. The average roughness (Ra) was calculated for each specimen. The group mean was then calculated for the 8 specimens.

Vickers microhardness testing

Immediately following polishing, the giomer or composite specimen was mounted on a hardness tester (Micromet 2130 Microhardness Tester, Buehler, Lake Bluff, IL, USA) to assess the VHN. A 500 g load was applied through a 136 ° pyramidal diamond indenter for 15 seconds. Three readings equally distributed over the surface, but away from the periphery of the sample, were taken on the top surface. The mean surface roughness (VHN) for each specimen was calculated. The group mean was then calculated for the 8 specimens. 

Simulated toothbrushing

Toothbrushing occurred in an automated tooth brushing simulator (Toothbrushing Simulator MEV 4X-3D, Odeme Dental Research, Luzerna, Brazil) at 37^o^C and brushed with a soft, manual nylon-bristled toothbrush (Wisdom Sensitive, Suffolk, England) using a toothpaste to water slurry in a ratio of 1:2. The toothpaste slurry was hand-mixed prior to application to the composite specimen. The specimens were brushed at two strokes per second with a load of 200 g for 420 and 2520 strokes to simulate one and six months of brushing, respectively. Ra and VHN readings were repeated as described in the preceding section.

Statistical analysis

Statistical analysis was performed using the Statistical Package for Social Sciences version 29 (IBM Inc., Armonk, New York). A repeated-measure two-way ANOVA with a significance level of p≤0.05 was used to determine statistical significance for each of the tested parameters of roughness (Ra) and microhardness (VHN). The type of toothpaste used was an included factor. The assumptions for use of the repeated measure two-way ANOVA, of normality and sphericity, were verified for each of the tested groups using Shapiro-Wilk, Box's Test of Equality of Covariances, and Mauchly's Test of Sphericity. Pairwise comparisons were facilitated using a post-hoc Bonferroni. Partial eta-squared values were examined to understand what percentage of the variance in Ra and VHN could be attributed to the toothpaste when toothpaste produced a significant change in these parameters.

## Results

The means and standard deviations of Ra and VHN for each of the materials are presented in Tables 2-7 

**Table 2:** Mean ± SD roughness values for polished giomer at baseline, one-month, and six-month intervals

Toothpaste		Time Interval	
	Baseline	1 month	6 months
Optic White	0.60 ± 0.35	0.65 ± 0.35	0.64 ± 0.27
Pursito	0.66 ± 0.28	0.90 ± 0.34	0.54 ± 0.29
Crest 3D w Charcoal	0.70 ± 0.28	0.79 ± 0.22	0.76 ± 0.37
Curaprox	0.78 ± 0.24	0.81 ± 0.22	0.80 ± 0.28
Colgate Essentials	0.75 ± 0.37	0.79 ± 0.16	0.61 ± 0.23

**Table 3:** Mean ± SD roughness values for polished nanofilled material at baseline, one-month, and sic-month intervals Two-way repeated measure ANOVA produced significant results for time*toothpaste (p-value=0.021, F-Value: 2.45)

Toothpaste		Time Interval	
	Baseline	1 month	6 months
Optic White	0.72 ± 0.51	0.63 ± 0.23	0.56 ± 0.24
Pursito	0.56 ± 0.20	0.75 ± 0.28	0.46 ± 0.19
Crest 3D w Charcoal	0.51 ± 0.13	0.58 ± 0.14	0.56 ± 0.21
Curaprox	0.80 ± 0.56	0.41 ± 0.16	0.64 ± 0.24
Colgate Essentials	0.58 ± 0.26	0.60 ± 0.27	0.65 ± 0.34

**Table 4:** Mean ± SD roughness values for polished nanohybrid material at baseline, one-month, and six-month intervals Two-way repeated measure ANOVA produced significant results for time (p=0.001, F-value: 7.98)

Toothpaste		Time Interval	
	Baseline	1 month	6 months
Optic White	0.73 ± 0.24	0.66 ± 0.26	0.55 ± 0.23
Pursito	0.74 ± 0.26	0.74 ± 0.27	0.50 ± 0.09
Crest 3D w Charcoal	0.49 ± 0.20	0.48 ± 0.27	0.40 ± 0.09
Curaprox	0.61 ± 0.28	0.59 ± 0.22	0.49 ± 0.12
Colgate Essentials	0.48 ± 0.15	0.64 ± 0.25	0.48 ± 0.10

**Table 5:** Mean ± SD microhardness values for polished giomer at baseline, one-month, and six-month intervals Two-way repeated measure ANOVA produced statistically significant results for time (p<0.001, F value: 20.35) and time*toothpaste (p=0.006, F value: 2.98)

Toothpaste		Time Interval	
	Baseline	1 month	6 months
Optic White	61.53 ± 11.9	71.79 ± 14.61	79.35 ± 15.43
Pursito	59.80 ± 7.23	62.76 ± 7.70	59.31 ± 5.80
Crest 3D w Charcoal	54.75 ± 7.20	66.18 ± 10.7	64.6 ± 6.26
Curaprox	54.50 ± 7.00	60.31 ± 6.39	62.00 ± 7.46
Colgate Essentials	59.53 ± 8.60	63.68 ± 8.67	63.91 ± 9.51

**Table 6:** Mean ± SD microhardness values for polished nanohybrid at baseline, one-month, and six-month intervals Two-way repeated measure ANOVA produced statistically significant results for time (p<0.001, F-value:12.9) and toothpaste (p=0.042, F-value: 2.78)

Toothpaste		Time Interval	
	Baseline	1-Month	6-Month
Optic White	89.35 ± 4.54	89.65 ± 5.05	98.98 ± 11.48
Pursito	89.91 ± 8.05	91.29 ± 6.55	90.86 ± 7.70
Crest 3D w Charcoal	93.80 ± 4.09	95.50 ± 6.05	100.14 ± 6.36
Curaprox	92.7 ± 3.10	95.71 ± 3.95	97.60 ± 2.21
Colgate Essentials	87.04 ± 3.70	88.59 ± 4.66	92.53 ± 9.33

**Table 7:** Mean ± SD microhardness values for polished nanofilled at baseline, one-month, and six-month intervals Two-way repeated measure ANOVA produced statistically significant results for time (p<0.001, F-value: 30.36)

Toothpaste		Time Interval	
	Baseline	1 month	6 months
Optic White	65.34 ± 6.27	69.39 ± 4.74	72.86 ± 8.20
Pursito	63.33 ± 4.20	67.51 ± 4.57	65.01 ± 4.40
Crest 3D w Charcoal	63.04 ± 5.82	67.51 ± 4.57	67.41 ± 4.99
Curaprox	63.33 ± 6.46	69.61 ± 5.89	71.89 ± 5.43
Colgate Essentials	63.96 ± 5.21	66.63 ± 5.52	67.76 ± 6.39

There were mixed results from baseline to six months for the various materials brushed with various toothpastes for the tested parameters of roughness (Ra), however, these results were not statistically different from each other at the various brushing intervals. There were no significant differences between the baseline measurements for VHN for any of the tested materials.

Surface roughness

For the giomer samples, neither the effect of time (p=0.055) nor time*toothpaste (p=0.448) produced a statistically significant change in roughness values from baseline. Toothpaste did not have a significant effect on the roughness of the giomer samples (p=0.659). There was a trend of increasing roughness between months one and six, which then decreased by month six when brushed with the control (non-charcoal-containing, Optic White) and charcoal-containing toothpaste. Pairwise comparisons of material groups brushed with the various toothpastes demonstrated no significant differences in mean Ra.

When the nanofilled material was considered, time(p=0.761) nor toothpaste (p=0.915) produced no significant increase in roughness from baseline measurements, however, time*toothpaste (p=0.021) produced a significant reduction in Ra, with partial eta squared values suggesting the toothpastes contributed 21.9% to the variance in the average roughness of the nanofilled samples. When means were examined from baseline to one-month and six-month intervals, a trend of decreasing roughness was observed. Pairwise comparisons of material groups brushed with the various toothpastes demonstrated no significant differences in mean Ra.

When Ra for the nanohybrid material was considered, toothpaste produced no significant change (p=0.115), time produced a significant change in roughness (p=0.001), while time*toothpaste produced no significant change (p=0.441), with partial-eta squared values suggesting the toothpaste contributed 10.3% to the variance in the average roughness values of the nano-hybrid samples. Except for nano-hybrid materials brushed with Colgate Essentials, there was a general trend of decreasing roughness over the six months of simulated brushing. Pairwise comparisons of material groups brushed with the various toothpastes demonstrated no significant differences in mean Ra.

Microhardness

Giomer samples showed no statistically significant difference in mean VHN due to toothpastes (p=0.56). When the factor of time was considered, there was a significant difference in HVN (p<0.001), and when the intercept of time*toothpaste was considered (p=0.006). The toothpastes contributed 22.6% (partial eta squared value) to the variance of the overall VHN of the giomer specimens. When pairwise comparisons were considered, there were significantly different mean VHN values between baseline and month one and baseline and month six. Pairwise comparisons of material groups brushed with the various toothpastes demonstrated no significant differences in mean VHN.

For the nanofilled material, there was a significant change in mean VHN values over time (p<0.001) but when the factor of the toothpaste (p=0.427) or the intercept of time*toothpaste was considered no significant change was noted (p=0.077), with the toothpaste contributing 17.7% to the variance the microhardness values of the nanofilled material. Both nanofilled and nanohybrid materials showed a trend of increasing hardness throughout simulated brushing. Pairwise comparisons of material groups brushed with the various toothpastes demonstrated no significant differences in mean VHN.

The nanohybrid material showed significant increases in VHN with time (p<0.001) and toothpaste (p=0.042). There were also significant differences in VHN due to the interaction of time*toothpaste (p=0.023), with partial eta-squared values suggesting the effect of the toothpaste accounting for 24.1% of the variance in VHN being attributed to the toothpaste. Pairwise comparisons of material groups brushed with the various toothpastes demonstrated no significant differences in mean VHN.

## Discussion

Average roughness (Ra) and microhardness (VHN) are important surface properties for esthetic dental materials, which can influence clinical longevity [8]. Abrasives commonly included in various toothpastes can alter the surface properties of esthetic restoratives [12,14]. The roughened surfaces of restorative materials can promote plaque adhesion and accumulation [15]. Surface roughness can also influence the color stability of the material [16]. Microhardness, or the ability of a material to resist indentation, is often used as an indirect measure of the degree of polymerization of the resin matrix [3] and can influence subsequent abrasive wear of the material from masticatory function, polishing, and toothbrushing [17]. Baseline microhardness measurements for giomer were similar to those of the nanohybrid and nano-filled material. The authors postulated that the tested giomer was comparable to both resin composite materials in terms of microhardness. This concurs with recent findings where the microhardness of bulk-filled giomer was comparable to other bulk-filled resin composites [18].

The trend of increasing microhardness measurements from baseline to six months for all the materials could be attributed to the toothpaste-driven removal of the oxygen-inhibited layer over the simulated time periods. Despite the samples being polished, this layer's remnants could still be present. The oxygen-inhibited layer varies in thickness from four to 40 micrometers and is often cited as having lower microhardness values [19]. The tribological mechanisms associated with wear of the composite surface because of continued brushing with toothpastes with abrasive particles would have eliminated this layer, exposing deeper layers of the polymerized composite with improved hardness values. This aligns with partial eta-squared values where toothpastes accounted for between 17.7 and 25.4% of the variance of VHN across the various materials.

There was an unexpected trend of either decreasing roughness or roughness values that were not statistically different from baseline to six months. This finding aligns with the work of Yilmaz et al., who demonstrated no significant differences in roughness values for any of the tested esthetic materials before and after brushing with whitening toothpastes [14]. However, these results contrast with recent work, which demonstrated increased roughness of esthetic restoratives when brushed with charcoal toothpastes at longer brushing intervals [12]. The interaction of time and toothpaste caused significantly lower roughness values for the nanofilled material. The authors attributed this to the equal wear rate by the abrasive particles of the various toothpastes of aggregated and agglomerated nanoparticles within the nanofilled composite. The exception to this trend of decreasing roughness was noted with samples of the nanofilled restorative brushed with Colgate Essentials and Crest 3D. Recent work has shown these dentifrice formulations to contain large agglomerated silica abrasive particles [20], which can worsen the abrasive potential of toothpastes that contain them [21].

Previous research has shown that nano-composites, when subjected to simulated brushing for longer periods of time, are characterized by rough surfaces statistically different from baseline [22]. For longer periods of brushing, the surface of bi-phasic restoratives is characterized by distinctive porosities on the surface of the materials and increased roughness. Nanohybrids, having larger particle sizes, demonstrate even rougher surfaces with simulated brushing compared to nano-filled materials [23]. It can be postulated that the relative proportion of the abrasive components in the various charcoal-containing formulations, including the positive control, Optic White, was insufficient to cause a characteristic expected increase in roughness in any of the tested materials over the periods of brushing utilized in this study. This aligns with partial eta-squared values where toothpastes accounted for between 10.2 and 25.4% of the variance of Ra across the various materials. Further research would investigate the effect of longer simulated brushing times with charcoal-containing toothpaste on the microhardness and roughness of the tested restoratives.

When brushed with toothpaste, the abrasive wear of the giomer and composites, classified as bi-phasic materials, involves the initial wear of the softer resin matrix, followed by the subsequent loss of harder filler particles as the filler particles are unsupported by the surrounding resin [22,24]. The nanofilled materials (Z350) have both non-agglomerated nanoparticles and agglomerated nanoclusters composed of nanoparticles. Simulated brushing over time, with toothpastes, would theoretically result in an equitable loss of individual nanoparticles or particles from nanoclusters. The mechanism of surface loss of resin, followed by nano-sized filler particles, would result in similar roughness values compared to baseline, as noted in this study. 

Limitations

The length of simulated brushing is the major limitation of this work, given the mixed results. Additionally, the restorative specimens were not artificially aged or thermocycled, which is important to mimic intra-oral conditions over time. Given these limitations, the current findings must be cautiously interpreted. Further work would include protocols for aging and thermocycling, and increasing the length of simulated brushing to ascertain if statistically significant changes in the surface properties of the tested materials occur from baseline, because of brushing with charcoal-containing toothpastes. Further research would include a qualitative assessment using scanning electron microscopy of the surfaces of the esthetic materials before brushing and at the end of simulated brushing

## Conclusions

Within the limitations of this study, the null hypothesis was rejected since the factors of time, toothpaste, or the interaction of time and toothpaste produced significantly different roughness and hardness values of the tested materials. There was a general trend of increasing VHN over time. Ra values either decreased or remained statistically similar from baseline to six months. Throughout simulated brushing, there were no differences in surface properties because of individual toothpaste formulations, with pairwise comparisons producing no statistically significant results. Given the limitations of this in vitro work, caution must be applied in extrapolating these findings to clinical situations. 
